# Supplementary material for: Equivariant electronic Hamiltonian prediction with many-body message passing
Source: NPJ Comput Mater. 2026 Mar 11;12(1):169. doi: 10.1038/s41524-026-02020-1 (PMC13111152; doi:10.1038/s41524-026-02020-1)
Supplement: Supplementary file 1 — Supplementary Information [file 41524_2026_2020_MOESM1_ESM.pdf]

Supplementary information for
Equivariant Electronic Hamiltonian Prediction with Many-Body Message Passing

Chen Qian[1], Valdas Vitartas[1,2], James R. Kermode[2], and Reinhard J. Maurer*[1,3,4]

[1]Department of Chemistry, University of Warwick, Coventry, CV4 7AL, United Kingdom
[2]Warwick Centre for Predictive Modelling, School of Engineering, University of Warwick, Coventry, CV4 7AL, United Kingdom
[3]Department of Physics, University of Warwick, Coventry, CV4 7AL, United Kingdom
[4]Faculty of Physics, University of Vienna, Vienna A-1090, Austria

*Correspondence: reinhard.maurer@univie.ac.at

Contents

Supplementary Note S1 Hamiltonian Matrix MAE Analysis **3**

Supplementary Note S2 Additional Gold Results **4**
Supplementary Note S2.1 Correlation Order vs Number of Layers 4
Supplementary Note S2.2 Learning Curves 4
Supplementary Note S2.3 Time-to-Solution Comparison 5

Supplementary Note S3 Benchmarking for molecular datasets **5**

Supplementary Note S4 Data Efficiency of the Model **5**

Supplementary Note S5 Time-to-Solution Comparison: DeepH-E3 and MACE-H **6**

Supplementary Note S6 Many-Body-Expansion for Different Interaction Range **6**
Supplementary Note S6.1 The Influence of the Many-Body Expansion for Bilayer Predictions 6
Supplementary Note S6.2 Atom Perturbation Analysis of Hamiltonian Model Response . 8

Supplementary Note S7 Shift-and-Scale Operation on the Last-Layer Irreps **10**
Supplementary Note S7.1 Numerical Instability for Large Matrix Value Ranges 10
Supplementary Note S7.2 Ablation Test for Shift-and-Scale Operation 13

Supplementary Note S8 The correlation between hermiticity error and prediction error **13**

Supplementary Note S9 Core Projection and Sparsification **16**
Supplementary Note S9.1 Reciprocal-to-Real Transformation Convergence 16
Supplementary Note S9.2 Eigenvalue Accuracy: 16

Supplementary Note S10 Additional data **18**

S1 Hamiltonian Matrix MAE Analysis

To compare the performance of MACE-H and DeepH-E3, we first trained a series of models on shifted Bi_2Te_3 bilayers and then evaluated them using shifted test data and twisted bilayers. Supplementary Fig. S1 shows the corresponding analytic MAE matrices. For performance on shifted bilayers, MACE-H outperformed DeepH-E3, with the average, maximum, and minimum of the error matrix being 0.28/0.48 meV, 2.82/2.95 meV and $7.98 \times 10^{-5}/3.59 \times 10^{-2}$ meV. In Supplementary Fig. S1a, the error matrix blocks (segmented by the red lines) between different spin channels are usually of similar magnitudes to the ones between the same spin channels for DeepH-E3. Supplementary Fig. S1c shows that the MACE-H (employing shift and scale operation) manifested much lower errors between different spin channels. Moreover, the improvement of MACE-H is also manifested in the error matrix between the same spin channels. When applied to the twisted bilayers, the average, maximum, and minimum of the error matrices of MACE-H and DeepH-E3 are 1.31/0.70 meV, 26.11/11.11 meV and $5.78 \times 10^{-5}/2.59 \times 10^{-2}$ meV with noticeably larger errors of MACE-H, as shown in Supplementary Fig. S1b, d.

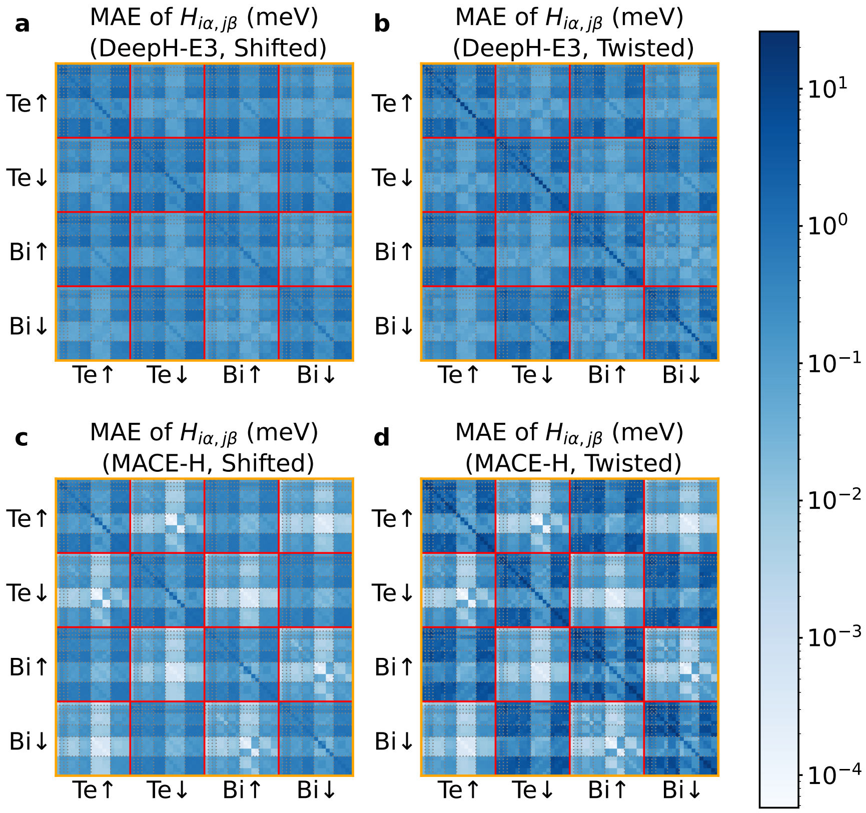

Figure S1: **Hamiltonian matrix element mean absolute error (MAE) comparison between MACE-H and DeepH-E3 for shifted and twisted Bi_2Te_3.** The MAE matrix of (**a**) shifted Bi_2Te_3 bilayer predicted with DeepH-E3, (**b**) twisted Bi_2Te_3 bilayer predicted with DeepH-E3, (**c**) shifted Bi_2Te_3 bilayer predicted with MACE-H, (**d**) shifted Bi_2Te_3 bilayer predicted with MACE-H. The MACE-H model in (**c**) and (**d**) includes shift-and-scale operations (see Supplementary Note S6).

S2 Additional Gold Results

S2.1 Correlation Order vs Number of Layers

In order to investigate the effect of increasing correlation order ν for the gold dataset, six different models were trained by varying the correlation order ν and the number of message-passing layers T as shown in Supplementary Fig. S2. The model used in the main text corresponds to $\nu = 2$ and $T = 2$ unless stated otherwise.

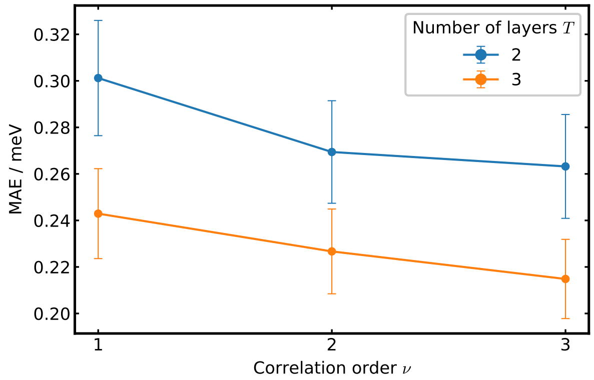

Figure S2: **Hamiltonian mean absolute error (MAE) for a range of models trained on the gold dataset with different correlation orders ν and number of layers T.** The error bars were obtained by computing the standard deviation $\sigma_{\nu,T}$ of the MAE across configurations in the test set, and plotting $\pm\sigma_{\nu,T}$ intervals.

S2.2 Learning Curves

To explore model convergence with respect to gold dataset size, MACE-H and DeepH-E3 models were trained with a range of training dataset sizes, and the MAE was computed for each model using the same test set as shown in Supplementary Fig. S3.

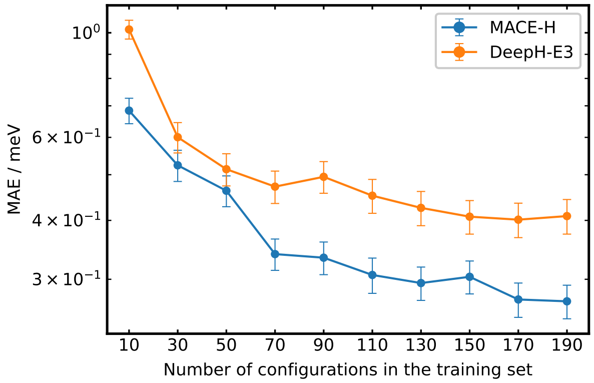

Figure S3: **Learning curves for MACE-H and DeepH-E3 for the gold dataset.** The reported error bars correspond to $\pm\sigma_{N_{\text{train}}}$, where $\sigma_{N_{\text{train}}}$ is the standard deviation of the MAE across configurations in the test set for each model trained on N_{train} configurations.

S2.3 Time-to-Solution Comparison

In order to benefit from the computational speedup offered by the machine learning model, training data containing self-consistent Hamiltonians needs to be generated. For the machine learning model to be practical, the computational speedup offered by the model needs to outweigh the initial cost of generating the training data.

Bulk gold data consists of 200 self-consistent Hamiltonian matrices, each of which on average took 4 hours to calculate with 32 processes on an AMD EPYC 7742 CPU. This results in about 128 CPUhs per matrix, or 25600 CPUhs for the whole dataset.

On the other hand, test set prediction of 10 configurations with MACE-H took 10 seconds per configuration with 8 CPU threads on an AMD EPYC 7742 CPU. The prediction would be even faster on a GPU as shown for 2D systems in the main manuscript (Fig. 5). The computational time taken per configuration is equal to 0.02 CPUh, which is three orders of magnitude faster than DFT.

For larger configurations, we could expect even larger speedup offered by the machine learning model. This is because self-consistency cycles in DFT scale as $O(N_{\text{at}}^3)$, whereas MACE-H prediction scales linearly with the number of atoms. However, this speedup would diminish if the predicted Hamiltonian was diagonalised using a dense eigensolver with an $O(N_{\text{at}}^3)$ scaling.

S3 Benchmarking for molecular datasets

Apart from the model assessment for the 2D material and bulk gold datasets, we also evaluated our MACE-H performance on the MD17 molecular datasets, including water, ethanol, malondialdehyde, and uracil. To make a fair comparison with previous models, we adopted the same training, validation, and test data split. While MSE loss is adopted for the 2D materials and bulk gold dataset, we employ the $loss = MAE + MSE$ for molecules, as also used by QHNet. The results on test sets using the MAE metric are shown in Supplementary Table S1. We can tell that the irreps-based equivariant GNNs (PhiSNet, QHNet, SPHNet, and our MACE-H) achieve higher accuracy than the covariant GNN (SchNorb) and the local-coordinate-based GNN (DeepH). Compared with these previous models, which employ 5 layers, our model requires only 3 convolution layers to achieve similar performance. The overall performance of these models is not only dependent on the model framework itself, but also sensitive to the choice of hyperparameters like learning rate or number of training steps. For example, in the existing literature, two different groups of results can be found for PhiSNet and QHNet (as in Supplementary Table S1), one using 200,000 steps for affordable training and the other adopting up to 1,000,000 steps for higher accuracy but with a much slower training process. Our MACE-H model strikes a good balance between the optimizing steps required and accuracy. The corresponding hyperparameters for the MACE-H model training of MD17 datasets (using the shift-and-scale operation) are in Supplementary Table S2 .

S4 Data Efficiency of the Model

To assess the effect of different hyperparameter settings on MACE-H data efficiency, we adopted different settings. In Supplementary Fig. S4, Deep-H uses spherical harmonics with l up to 4, MACE-H uses the same settings in the edge-update block and specifies the node-wise MPNN block with spherical harmonics with l up to 4, and hidden states with azimuthal number up to 2. The higher setting of MACE-H uses spherical harmonics l up to 5 and hidden state azimuthal numbers up to 5. Models with two ($T = 2$) and three layers are compared. The correlation order $\nu = 3$

Table S1: MAE (in meV) of matrix elements on MD17 in comparison with other models. For the PhiSNet and QHNet, 5 convolutional layers are used, while MACE-H only uses 3 layers. Due to the high training overhead of TPs, previous literature reports two groups of results for PhiNet and QHNet, one using 200,000 steps for affordable training (the value before the slash symbol) and the other adopting up to 1,000,000 steps for higher accuracy but with a much slower training process (the value after the slash symbol).

Dataset	SchNorb	DeepH	PhiSNet	QHNet	SPHNet	MACE-H (ours)
Water	4.501	1.048	0.426/0.479	0.282/0.294	0.631	0.512
Ethanol	5.099	0.601	0.331/0.547	0.348/0.569	0.572	0.412
Malondialdehyde	5.200	0.547	0.335/0.580	0.326/0.562	0.586	0.349
Uracil	6.199	0.470	0.292/0.507	0.271/0.547	0.526	0.403

Table S2: Hyperparameters of the MACE-H models for the MD17 datasets. The e_{N}, l_r, N_B, N_{layers}, ν, L_{MACE}, L_{node}, L_{hidden}, and L_{edge} stand for the node-update radial basis (B for Bessel and G for Gaussian), learning rate, batch size, number of layers, correlation order, the largest azimuthal number of the intermediate MACE block irreps, the intermediate node-wise block irreps, the many-body expansion hidden states irreps, and the intermediate edge-wise block irreps.

Dataset	Train/val/test data split	e_{N}	l_r	N_B	epochs	N_{layers}	ν	L_{MACE}	L_{hidden}	L_{edge}
Water	500/500/3900	G	0.01	10	2000	3	3	4	4	4
Ethanol	25000/500/4500	G	0.01	64	1200	3	3	4	4	4
Malondialdehyde	25000/500/1478	G	0.01	64	1000	3	3	4	4	4
Uracil	25000/500/4500	G	0.01	64	1000	3	3	4	4	4

for all the MACE models. Benefiting from the higher expressive power of many-body expansion, MACE-H shows higher accuracy compared to DeepH-E3. While increasing the model depth helps to increase the data efficiency, the tighter setting of the many-body-expansion with larger correlation order ν and minimum azimuthal number L_{max} of hidden states seems to be more effective for higher accuracy across different training data sizes. The overall Bi_2Te_3 dataset contains 256 configurations.

S5 Time-to-Solution Comparison: DeepH-E3 and MACE-H

To compare the inference time between DeepH-E3 and MACE-H, we perform tests using systems of various sizes in Supplementary Fig. S5. The inference time is obtained as time-to-solution using a single sample batch. Since the most computationally costly operation is the edge-wise update, the overall inference time of MACE-H is comparable to DeepH-E3 as they share the same edge-update block. No significant overhead due to the many-body expansion and the node degree expansion block can be identified.

S6 Many-Body-Expansion for Different Interaction Range

S6.1 The Influence of the Many-Body Expansion for Bilayer Predictions

Supplementary Fig. S6a shows the performance of the model that was trained on shifted bilayers applied to twisted bilayer structures. While we found MACE-H predictions to be more accurate than DeepH-E3 for shifted bilayers, MACE-H either provides similarly accurate or slightly less

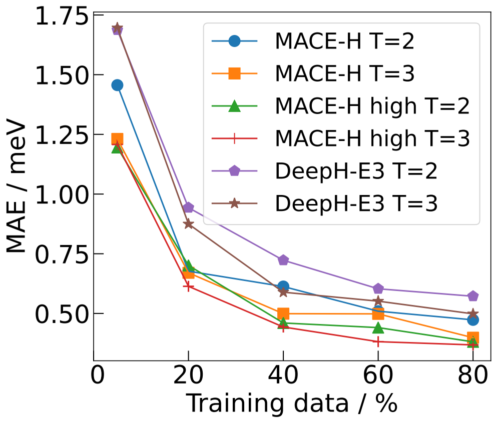

Figure S4: **Test set MAE for DeepH-E3 and MACE-H with different settings for shifted SOC $\mathbf{Bi_2Te_3}$ bilayers.** The label "high" means higher settings of spherical harmonics and the hidden states of many-body expansion, T, stand for model depth. The correlation order is $\nu = 3$ for all the MACE-H models. The overall shifted SOC Bi_2Te_3 bilayers dataset contains 256 configurations.

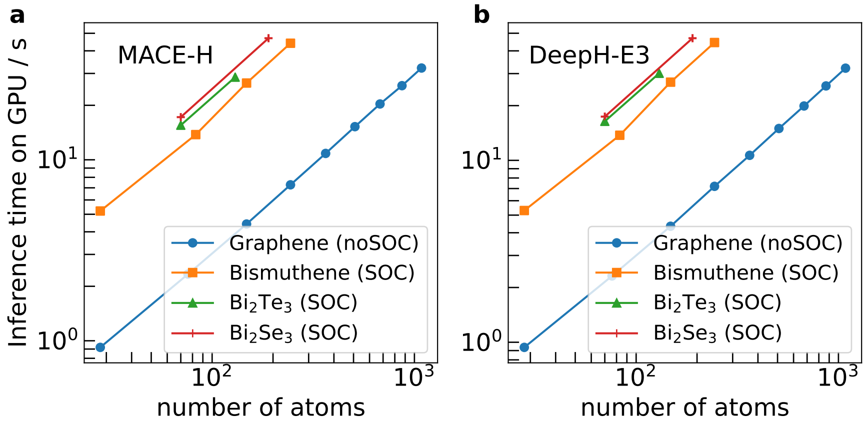

Figure S5: **Inference time comparison between (a) MACE-H and (b) DeepH-E3 on a single A100 GPU.**

accurate predictions for the twisted bilayers. We note that the Bi_2Se_3 dataset is three times larger than Bi_2Te_3. Since shifted and twisted bilayers have similar atomic environments, the difference may arise from differences in the range of interactions. To study the influence of the many-body expansion on the shifted and twisted bilayers, we evaluate models with different settings of the many-body expansion block in Supplementary Fig. S6b. It should be noted that to make a fair comparison with DeepH-E3, the MACE-H model used here does not incorporate the shift-and-scale operation. It shows that a tighter setting (using larger correlation order ν and the maximal azimuthal number of hidden states $L_{\max}$) of the many-body expansion will favour the performance on shifted bilayers in the test set but will bring further error for twisted bilayers, indicating the locality preference. This effect is even more distinguishable for the maximum values of the Hamiltonian MAE matrices in Supplementary Fig. S7, with the confidence interval showing the statistical significance. For predicting twisted bilayers with shifted bilayers as training data, it is recommended to use lower settings of ν and $L_{\max}$, and a larger dataset.

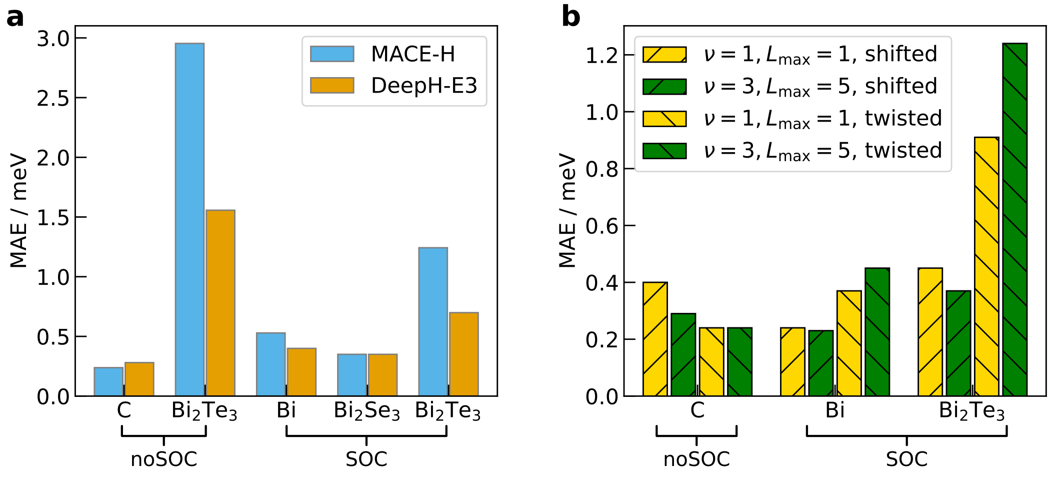

Figure S6: **Effect of correlation order for MACE-H predictions.** (**a**) Out-of-distribution MAE of matrix elements when applying the model trained on shifted bilayers to twisted bilayers for MACE-H and DeepH-E3. The correlation order $\nu = 3$ is used for MACE-H. (**b**) Test set (shifted systems) and out-of-distribution (twisted systems) MAE of matrix elements using different settings of correlation order ν and $L_{\max}$ in the many-body expansion.

To study the effect of correlation order, ν, and maximal azimuthal number of many-body expansion hidden states, $L_{\max}$, Fig. S8 shows the MAE using different hyperparameter settings. The other settings here are the same as those used in Supplementary Fig. S4, i.e., we used spherical harmonics with l up to 4, hidden states with azimuthal number ($L_{\max}$) up to 2, and correlation order $\nu = 3$. To avoid excessive computational overhead, we only used 20% of the dataset as training samples, and no hyperparameter tuning was used with only fixed hyperparameters. The right panel shows the tendency for preferred locality with larger ν and $L_{\max}$.

S6.2 Atom Perturbation Analysis of Hamiltonian Model Response

To further study the interaction range captured by MACE-H, we analyse how MACE-H predicted on-site matrix blocks at varying distances from a single atom change upon the displacement of this atom. We studied the response magnitude by the perturbation for the shifted (indexed by 72-0 in the dataset) and twisted (indexed 1-2 and 1-3) Bi_2Te_3 bilayer using DFT, MACE-H, and

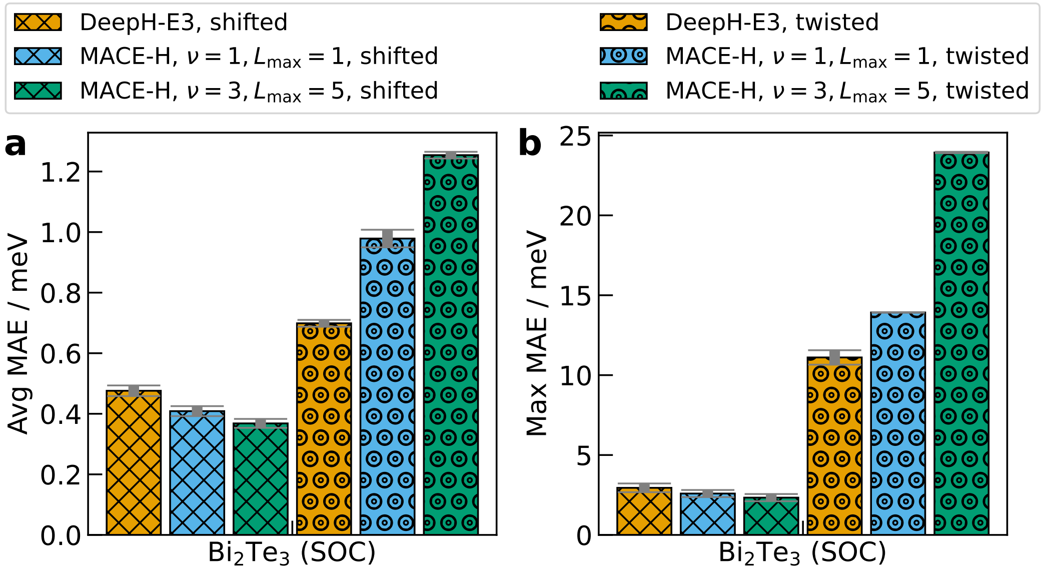

Figure S7: **(a) Average and (b) maximum values of the Hamiltonian error matrices.** Data is shown for shifted and twisted Bi_2Te_3 bilayer using DeepH-E3 and MACE-H with different settings of correlation order ν and maximum azimuthal number of hidden state $L_{\max}$. The confidence interval of a standard deviation $\sigma_{\nu,L_{\max}}$ is taken across the error matrix metric of individual samples.

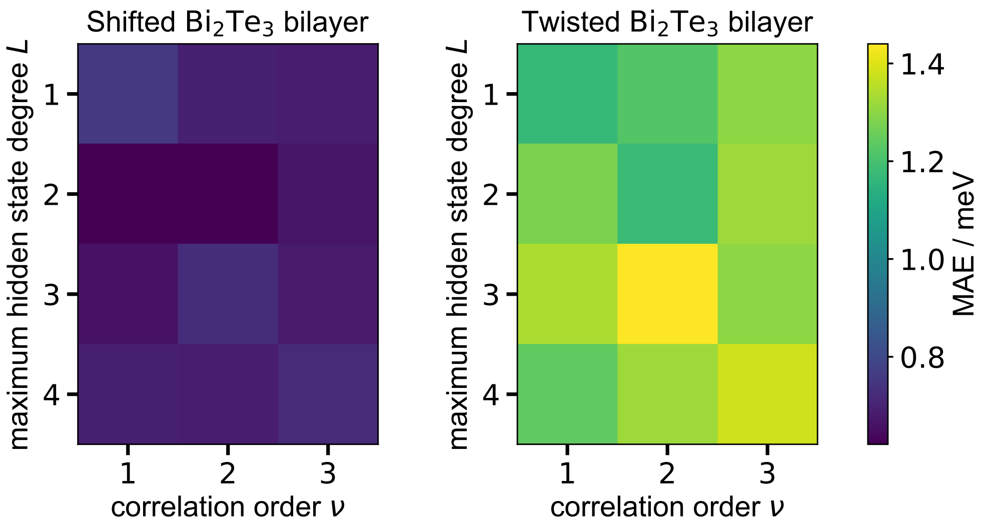

Figure S8: **The MAE for different settings of many-body expansion for shifted and twisted Bi_2Te_3 bilayers.**

DeepH-E3. Supplementary Fig. S9 shows the comparison between the shifted 72-0 and the twisted 1-2 unit cells using different methods. To further exclude the image interactions, we also construct the $2 \times 2 \times 1$ supercell counterparts of the unit cells, and the result also shows a similar observation. The data for the second twisted geometry shown in Supplementary Fig. S10 shows slightly different interaction range dependence of the models than the geometry shown in Figure 6 of the main manuscript, although the overall trends are the same.

For the shifted 71-0 bilayer unit cell, the overall onsite response error using MACE-H and DeepH-E3 are 9.6×10^{-2} and 13.3×10^{-2} meV, respectively. For the shifted 1-2 unit cell, the overall onsite response error using MACE-H and DeepH-E3 are 20.2×10^{-2} and 13.8×10^{-2} meV, respectively. However, for the supercell counterparts, the difference between MACE-H and DeepH-E3 regarding response error becomes negligible, with MACE-H showing a slightly lower error. We find that the change for supercells is mostly because MACE-H manifests lower response errors compared to DeepH-E3 for distances beyond 15 Å in the twisted supercells, which is a result of faster attenuation of the predicted response magnitude for longer distance regions, also showing the locality. Nevertheless, the larger matrix MAEs of twisted bilayers for MACE-H also exist in the supercells.

Here, the response to the perturbation ΔH_{ij} is defined as:

$$\Delta H_{ij} = H_{ij}^{\text{perturbed}} - H_{ij}^{\text{pristine}} \tag{S1}$$

where $H_{ij}^{\text{perturbed}}$ and H_{ij}^{pristine} are the matrix blocks between atom i and j of the perturbed and pristine conformation. The response magnitude is defined as the mean absolute value of the response matrix elements:

$$\|\Delta H_{ij}\| = \frac{1}{N_{orb}^2} \sum_{k_1=1}^{N_{\text{orb}}} \sum_{k_2=1}^{N_{\text{orb}}} |\Delta H_{ij}|_{k_1 k_2} \tag{S2}$$

where k_1, k_2 indicate the indices of the matrix element, N_{orb} is the number of orbitals in a Hamiltonian matrix block. The response error is defined as:

$$Error_{\Delta H_{ij}} = \frac{1}{N_{orb}^2} \sum_{k_1=1}^{N_{\text{orb}}} \sum_{k_2=1}^{N_{\text{orb}}} \left| \Delta H_{ij}^{\text{pred}} - \Delta H_{ij}^{DFT} \right|_{k_1 k_2} \tag{S3}$$

where $\Delta H_{ij}^{\text{pred}}$ and ΔH_{ij}^{DFT} are the response matrices using machine learning model prediction and DFT calculation, respectively.

S7 Shift-and-Scale Operation on the Last-Layer Irreps

S7.1 Numerical Instability for Large Matrix Value Ranges

Due to the large norm difference between the irreps of different element-orbital-pair resolved sub-blocks, adjusting the output accordingly may have the potential to accelerate the model convergence and increase the accuracy. However, irreps for different sub-blocks exhibit enormous differences over many orders of magnitude, which is a much more challenging scenario than energy predictions in machine learning interatomic potentials. For example, Supplementary Fig. S11b shows that when we directly apply the shift-and-scale operation, the learning curve oscillates, the model fails to converge well, and the overall accuracy deteriorates. This is because as we scale the norms of the last layer irreps in the feed-forward process, it not only pushes the result closer to the magnitude of the target but also will scale the according gradients again in the backpropagation

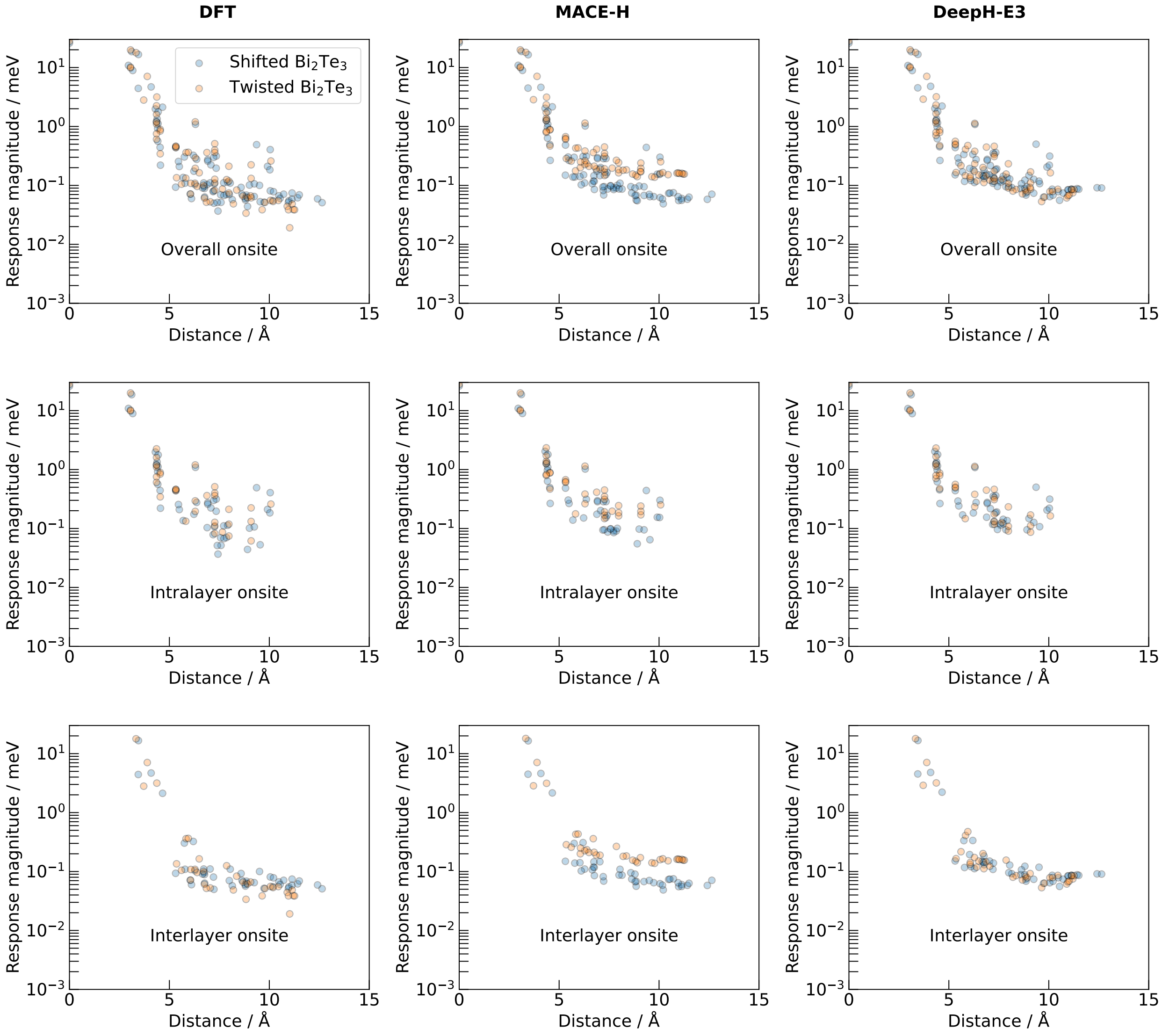

Figure S9: **Comparison of the predicted perturbation response magnitude between shifted (indexed by 72-0 in the dataset) and twisted (indexed by 1-2 in the dataset) unit cell bilayers using DFT, MACE-H, and DeepH-E3.**

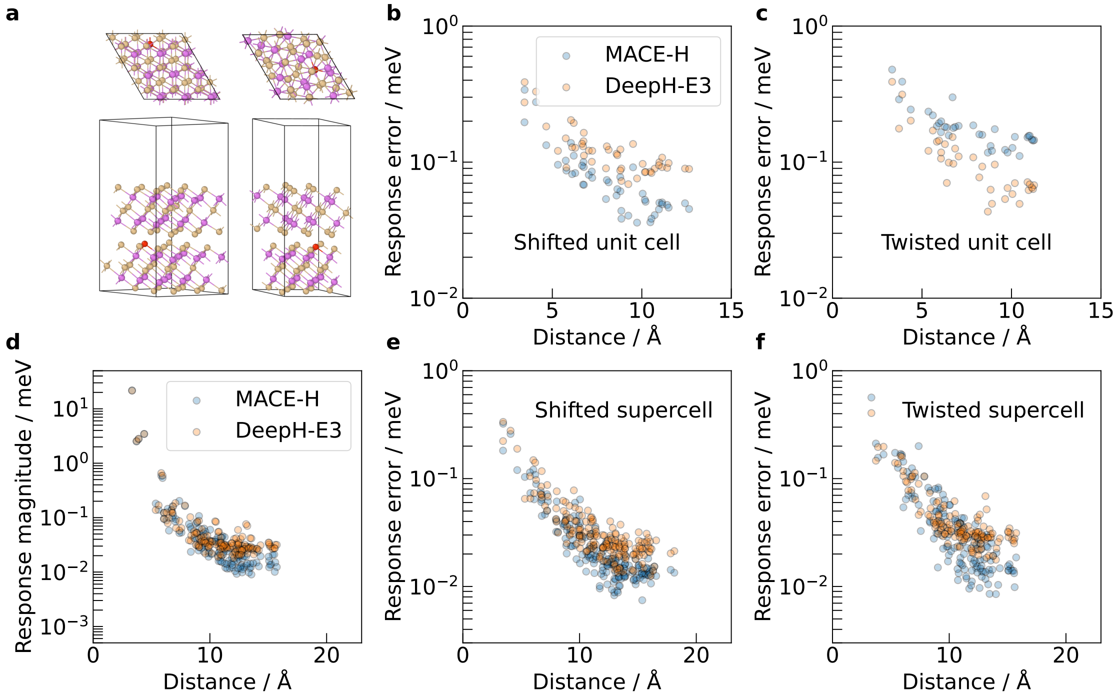

Figure S10: **Locality analysis of MACE-H compared to DeepH-E3 for the twisted bilayer based on single-atom displacement perturbation.** (**a**) The configuration of shifted (indexed by 72-0 in the dataset) and twisted (indexed by 1-2 in the dataset) Bi_2Te_3 bilayer unit cells with the red atom being the perturbed Te atom. (**b**) The response error of onsite matrix blocks w.r.t. the distance from the perturbed atom for a shifted bilayer unit cell. (**c**) The onsite response error for a twisted bilayer unit cell. (**d**) The decay rate comparison for the 2×2×1 twisted bilayer with varying distance using MACE-H and DeepH-E3. The onsite response error for the 2×2×1 shifted (**e**) and twisted (**f**) supercell counterparts. The onsite block components plotted in the figure are of different layers from the perturbed atoms.

process, which will induce gradient explosion (ascribed to irreps with larger standard deviation) and disappearance (ascribed to the components with smaller standard deviation). Furthermore, the components with larger standard deviations will also have their errors magnified at early epochs after the scaling operation, which further contributes to the numerical instability. To tackle the issue, we first attempted to set the standard deviation components with magnitude lower than 1 to 1 (Supplementary Fig. S11c). This method alleviates the instability in the earlier stage, but the large oscillations persist in the later stage. Moreover, manually trimming the standard deviation with a threshold will not be suitable for a streamlined workflow since it is inconvenient to determine the optimal threshold. Thus, we adopted the method to allow the scaling operation in the feed-forward process but to sidestep the scaling factors in the gradient backpropagation process by redirecting the gradient flow:

$$o_{ij,klm} = o^{(t)}_{ij,klm} + (o^{(t)}_{ij,klm}(\sigma_{z_{i,j},k} - 1)).detach() + \mu_{Z_{i,j},k} \tag{S4}$$

where $\mu_{Z_{i,j},k}$ and $\sigma_{z_{i,j},k}$ stand for the precalculated standard deviations and mean values of the corresponding target irreps, and the mean values will be set to zero for non-scalar vectors, the detach means that the gradient will be truncated for that term. Although the learning curve still has one observable oscillation at earlier epochs, the later stage converges faster than other methods with improved accuracy.

S7.2 Ablation Test for Shift-and-Scale Operation

To evaluate the effect by shifting and scaling the last layer according to the sub-block type resolved value distribution (i.e., the mean values for scalars and standard deviation for both scalars and vectors), we compared the average values of the MAE matrix using DeepH-E3, the pristine MACE-H, and the MACE-H with shift-and-scale operation. In Supplementary Fig. S12a, the shift-and-scale operation of MACE-H reduced the average MAE error from 0.32 meV of the pristine one to 0.27 meV. Due to the small magnitude of the SOC-involved matrix sub-blocks between different spin channels, the corresponding minimum value of the error matrix decreases by 3 magnitudes. A similar trend can also be observed for bulk Au in Supplementary Fig. S12b.

S8 The correlation between hermiticity error and prediction error

To further justify the correlation between the hermiticity error and the prediction error, which can be useful for future active learning methods, we first compared the median numbers of the prediction error of the matrix blocks with different quantiles of the hermiticity error in Supplementary Fig. S13. For the matrix blocks that have higher hermiticity errors in the ranking (lower quantile value), the corresponding prediction errors (measured by the median number of the corresponding matrix blocks) are usually higher than those with lower hermiticity errors.

Moreover, since the new label selection during active learning in practice is usually based on the entire geometry rather than single matrix block components, we compared the conformation-wise prediction error with the hermiticity error for models trained with varying training data sizes in Supplementary Fig. S14. The configuration-wise hermiticity errors are calculated as the mean of the block-wise errors within each configuration. For models trained with different numbers of conformations, the conformation-wise prediction errors are positively correlated with the hermiticity error, demonstrating the universality of this observation. This offered the possibility for an efficient measurement for active learning.

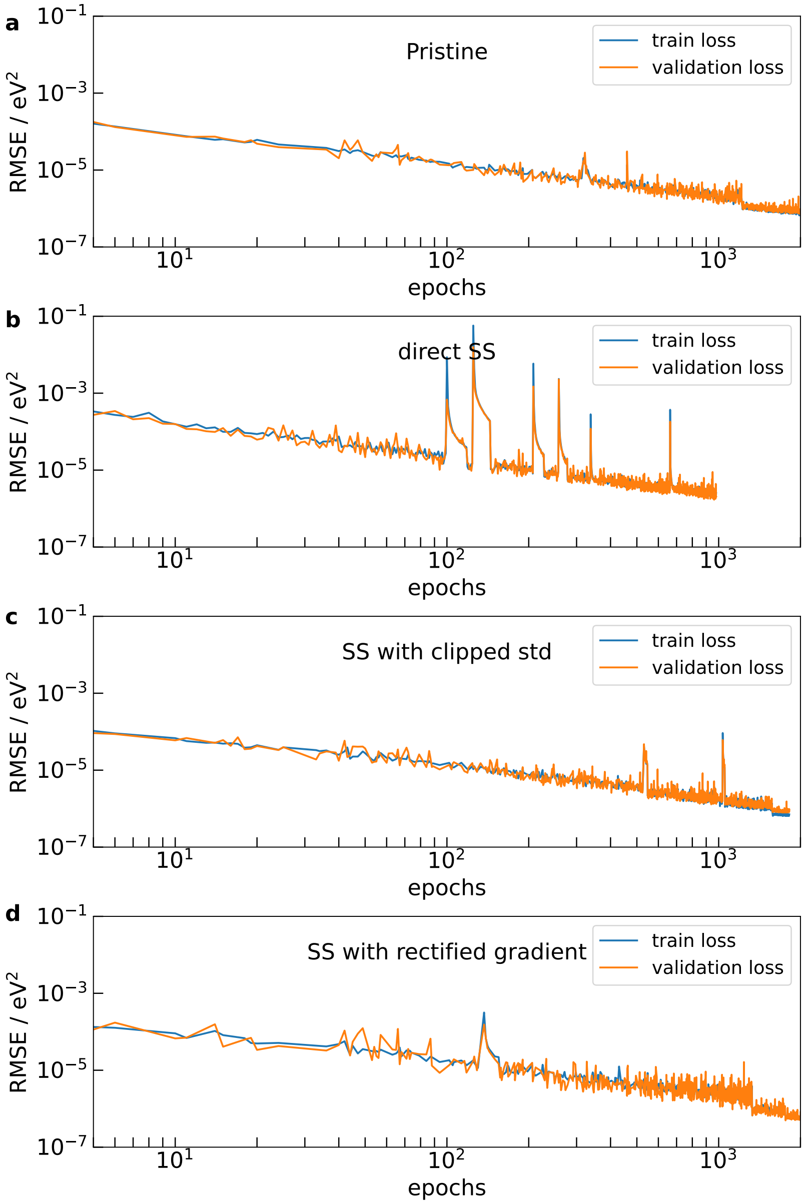

Figure S11: **The learning curve convergence of the models for shifted Bi_2Te_3.** (**a**) The original model training without shift-and-scale operation. (**b**) Direct application of the shift-and-scale operation to the last layer irreps. (**c**) Application of the shift-and-scale to the irreps with standard deviations of the norms lower than 1 manually set to 1. (**d**) Application of the shift-and-scale operation with a gradient bypass of the standard deviation in the backpropagation process.

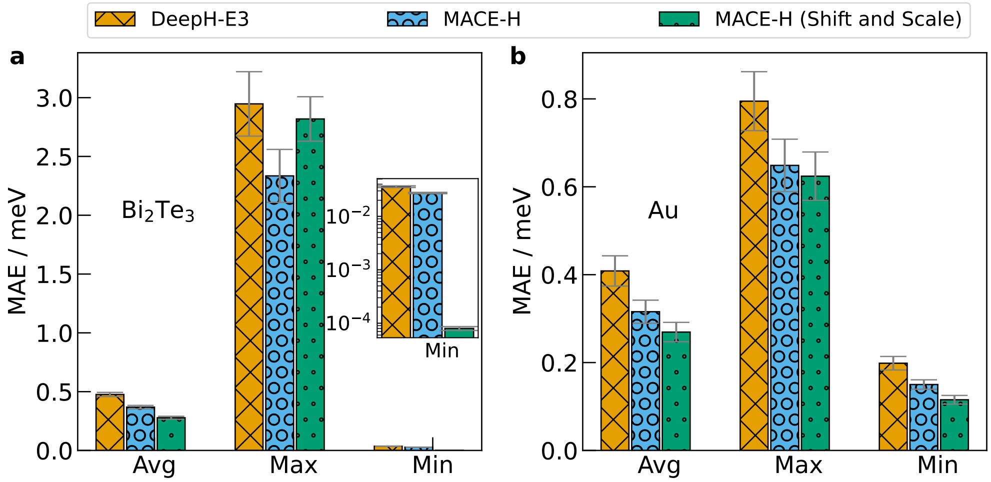

Figure S12: **Average values of the Hamiltonian error matrices.** Data shown for (**a**) Bi_2Te_3 bilayer and (**b**) bulk Au using DeepH-E3, the pristine MACE-H, and the MACE-H with the shift-and-scale operation. The confidence interval of a standard deviation σ is taken across the error matrix metric of individual samples. The inset in **a** is the magnified view of the Min MAE for Bi_2Te_3

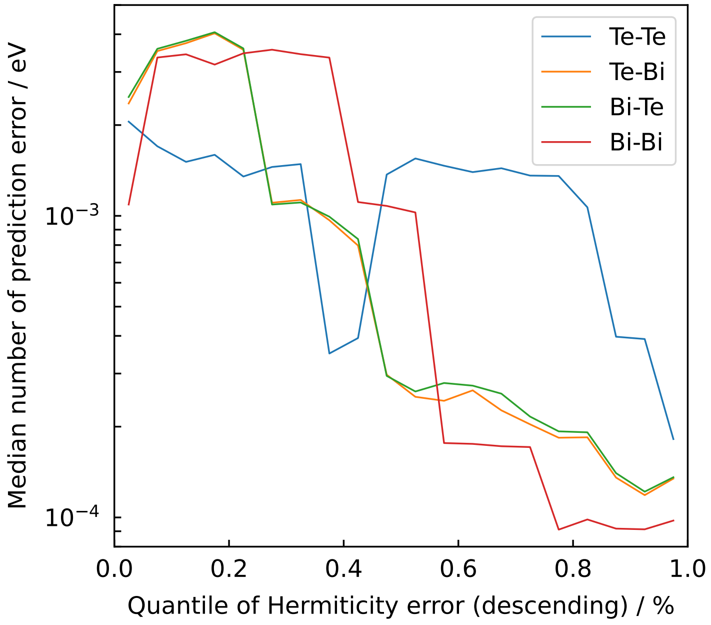

Figure S13: **The comparison of the median number of prediction errors for matrix blocks with corresponding quantile of hermiticity error in the twisted Bi_2Te_3 configuration resolved for different element pairs.** The quantile values here are in descending order, i.e., lower quantiles indicate higher hermiticity error.

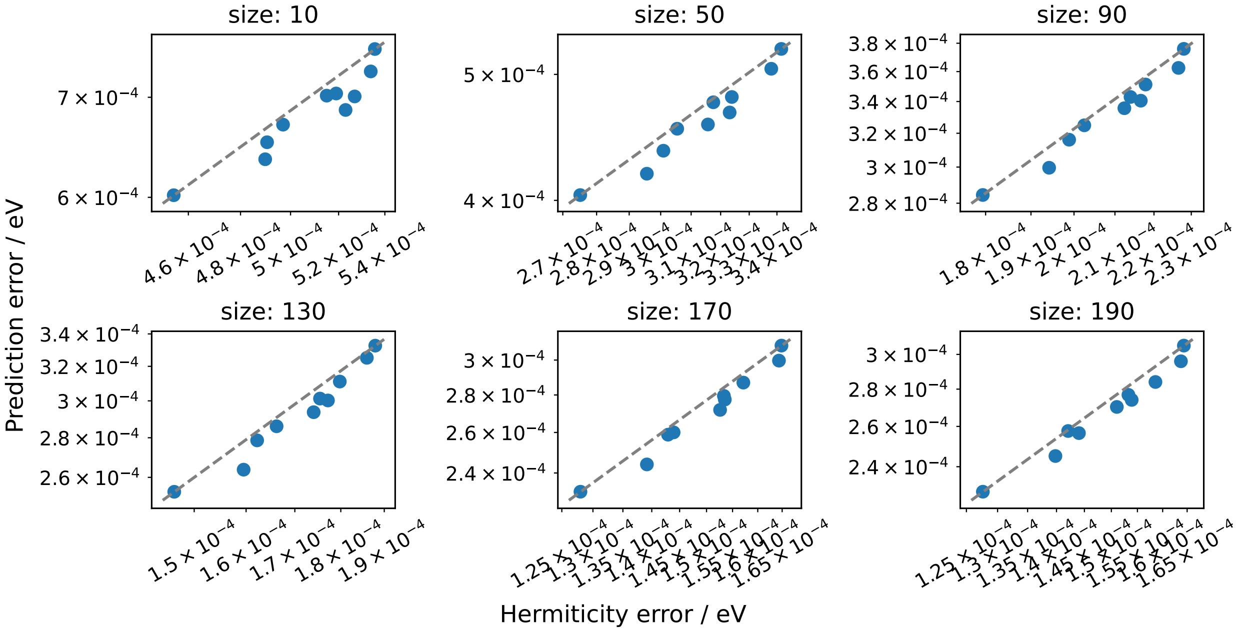

Figure S14: **The configuration-wise prediction error versus hermiticity error of held-out configurations for the gold models trained with varying training data sizes (as denoted by "size" in the subplots).** The configuration-wise hermiticity errors are calculated as the mean of the block-wise errors within each configuration.

S9 Core Projection and Sparsification

S9.1 Reciprocal-to-Real Transformation Convergence

It was investigated how many k-points are required to accurately perform the inverse Fourier transform from reciprocal to real space for core-projected Hamiltonian and overlap matrices, as shown in Supplementary Fig. S15. The convergence study was performed for a single configuration in the training set called 'run_Aims_duy8hd_p', which can be found on NOMAD. Based on these results, it was decided to perform core projection for the whole gold dataset using the 6x6x6 k-point grid. Note that the reciprocal cell for each configuration in the gold dataset is the same and contains reciprocal-space vectors of equal length.

S9.2 Eigenvalue Accuracy:

Core states from Hamiltonian and overlap matrices in the gold dataset were projected out as discussed in the main text and Sec. S9.1. Furthermore, to decrease the computational cost during training and inference, the off-site matrix blocks corresponding to interactions spanning over 10 Å were set to zero. This was motivated by the fact that absolute Hamiltonian and overlap matrix elements for edges beyond 10 Å become too small to significantly affect Hamiltonian eigenvalues as quantitatively discussed below.

It was examined how accurate the resulting eigenvalues from processed (core-projected and sparsified) matrices are compared to eigenvalues obtained from original full-basis matrices. This was investigated for a single gold configuration in the training set ('run_Aims_duy8hd_p'). It was found that the eigenvalue and electronic entropy errors at 1000 K between the full and processed matrices are equal to 1.41×10^{-4} eV and 6.96×10^{-12} eV K^{-1} Å^{-3}, respectively. These values are

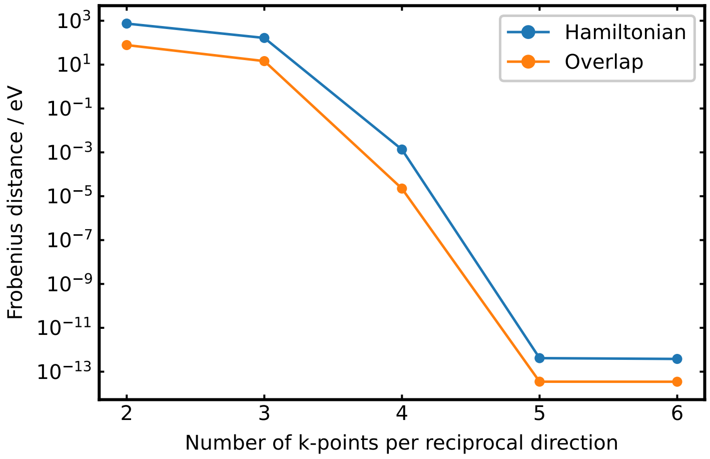

Figure S15: **Convergence of reciprocal-to-real transformation (inverse Fourier transform) as a function of k-point grid for core-projected Hamiltonian and overlap matrices for one of the configurations in the training set.** The Frobenius distance corresponds to $\|\boldsymbol{H}_{n_k} - \boldsymbol{H}_{n_{k,\mathrm{ref}}}\|_{\mathcal{F}}$, where $n_{k,\mathrm{ref}}$ is the reference number of k-points per reciprocal dimension, which was set to 7, corresponding to a $7 \times 7 \times 7$ k-point grid.

much smaller than the model errors shown in Supplementary Fig. 4 in the main text. The valence electronic band structures using full and processed matrices are shown in Supplementary Fig. S16

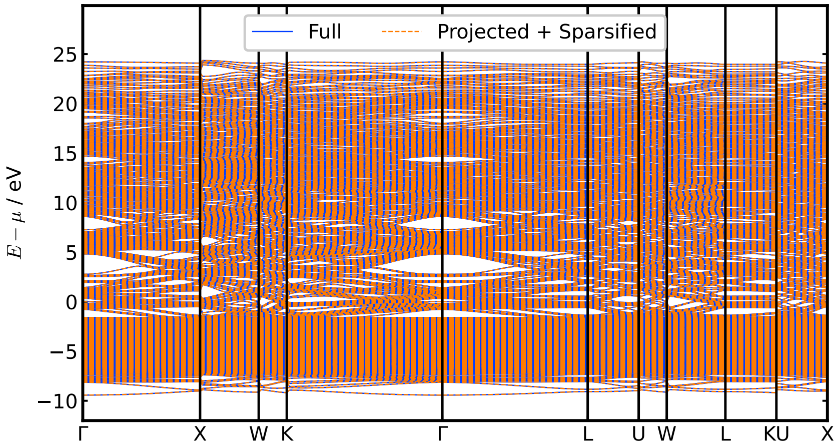

Figure S16: **Valence electronic band structure using full and processed (core-projected + sparsified) matrices for one of the configurations in the training set.** The bands from full and processed matrices are shifted with respect to their chemical potentials μ obtained with Fermi-Dirac smearing at 1000 K.

S10 Additional data

All data below (except where stated otherwise) employs MACE-H models without shift-and-scale operation.

Table S3: Hyperparameters of the models for different datasets in Fig. 4. The e_{N}, l_r, N_B, N_{layers}, ν, L_{MACE}, L_{node}, L_{hidden}, and L_{edge} stand for the node-update radial basis (B for Bessel and G for Gaussian), learning rate, batch size, number of layers, correlation order, the largest azimuthal number of the intermediate MACE block irreps, the intermediate node-wise block irreps, the many-body expansion hidden states irreps, and the intermediate edge-wise block irreps.

Dataset	MACE-H								DeepH-E3					
	e_{N}	l_r	N_B	N_{layers}	ν	L_{MACE}	L_{hidden}	L_{edge}	e_{N}	l_r	N_B	N_{layers}	L_{node}	L_{edge}
monolayer graphene (noSOC)	G	0.006	1	3	3	5	5	5	G	0.003	1	3	5	5
monolayer MoS_2 (noSOC)	G	0.006	1	3	3	5	5	5	G	0.005	1	3	5	5
bilayer graphene (noSOC)	G	0.003	1	3	3	5	5	5	G	0.003	1	3	5	5
bilayer Bismuthene (SOC)	G	0.01	1	3	3	5	5	5	G	0.005	1	3	5	5
bilayer Bi_2Se_3 (SOC)	G	0.01	1	3	3	5	5	5	G	0.005	1	3	5	5
bilayer Bi_2Te_3 (SOC)	G	0.008	2	3	3	5	5	5	G	0.004	2	3	5	5
bilayer Bi_2Te_3 (noSOC)	G	0.008	2	3	3	5	2	4	G	0.004	2	3	5	5
bulk Au (noSOC)	B	0.008	2	2	2	5	2	4	G	0.008	2	2	4	4

Table S4: MAE (in meV) for Hamiltonian matrix prediction on 2D monolayers without SOC using DeepH, DeepH-E3 and our MACE-H. The basis sets used for both graphene and MoS_2 are up to d-orbital.

model	Graphene (noSOC)	MoS_2 (noSOC)			
	C-C	Mo-Mo	Mo-S	S-S	overall
DeepH	2.1	1.3	0.9	0.7	0.95
DeepH-E3	0.27	0.51	0.44	0.36	0.45
MACE-H (ours)	**0.21**	**0.42**	**0.39**	**0.32**	**0.39**

Table S5: MAE (in meV) for Hamiltonian matrix prediction on 2D bilayers using DeepH, DeepH-E3 and our MACE-H. The basis sets are up to d-orbital, while the graphene and Bi_2Te_3 dataset doesn't involve SOC, the Bismuthene, Bi_2Te_3, Bi_2Se_3 involve SOC.

model	Graphene (noSOC)		Bi_2Te_3 (noSOC)		Bismuthene (SOC)		Bi_2Te_3 (SOC)		Bi_2Se_3 (SOC)	
	shifted	twisted	shifted	twisted	shifted	twisted	shifted	twisted		
DeepH	1.9	0.62	/	/	/	/	/	/	/	/
DeepH-E3	0.40	0.28	0.86	**1.56**	0.26	**0.40**	0.48	**0.70**	0.40	0.35
MACE-H (ours)	**0.31**	**0.24**	**0.58**	2.50	**0.23**	0.45	**0.37**	1.25	0.34	0.35

Table S6: MAE (in meV) on bilayer Bi_2Te_3 with SOC using different settings of radial basis and many-body expansion and its relation to the locality. The values in the parentheses represent the maximum value of the MAEs among different element pair-resolved orbital pairs, indicating the model performance for the most challenging orbital pair, which is the same as that in the following Tables S5 and S6. The B and G in parentheses stand for Bessel and Gaussian radial basis set, respectively.

model	shifted Bi_2Te_3 (SOC)				twisted Bi_2Te_3 (SOC)			
	Te-Te	Te-Bi	Bi-Bi	overall	Te-Te	Te-Bi	Bi-Bi	overall
DeepH-E3	0.63 (3.23)	0.48 (2.34)	0.41 (2.08)	0.50 (3.23)	0.90 (11.84)	0.59 (4.74)	0.66 (7.11)	0.68 (11.84)
MACE-H (B, v=3, L=2)	0.51 (3.08)	0.37 (1.84)	0.34 (1.75)	0.40 (3.80)	1.26 (17.91)	1.10 (12.10)	1.51 (23.83)	1.24 (23.83)
MACE-H (G, v=3, L=5)	0.46 (2.33)	0.35 (1.66)	0.30 (1.62)	0.37 (2.33)	1.26 (17.91)	1.10 (12.10)	1.51 (23.83)	1.24 (23.83)
MACE-H (G, v=3, L=2)	0.50 (2.49)	0.38 (1.80)	0.34 (1.66)	0.40 (2.49)	1.16 (18.11)	1.06 (9.19)	1.48 (20.93)	1.19 (20.93)
MACE-H (G, v=1, L=1)	0.57 (2.87)	0.43 (2.04)	0.38 (1.89)	0.45 (2.87)	1.03 (12.70)	0.82 (6.42)	0.98 (12.46)	0.91 (12.70)

Table S7: MAE (in meV) on bilayer Bi_2Se_3 with SOC using different settings of radial basis and many body expansion and its relation to the locality. The values in the parentheses show the maximum error of the Hamiltonian matrix elements. The B and G in parentheses stand for Bessel and Gaussian radial basis set, respectively.

model	shifted Bi_2Se_3 (SOC)				twisted Bi_2Se_3 (SOC)			
	Se-Se	Se-Bi	Bi-Bi	overall	Se-Se	Se-Bi	Bi-Bi	overall
DeepH-E3	0.39 (1.45)	0.45 (1.72)	0.32 (1.97)	0.40 (1.97)	0.37 (1.58)	0.38 (1.71)	0.30 (2.29)	0.35 (2.29)
MACE-H (B, v=3, L=2)	0.35 (1.33)	0.39 (1.57)	0.28 (1.69)	0.35 (1.69)	0.38 (1.95)	0.39 (1.84)	0.28 (2.41)	0.35 (2.41)
MACE-H (G, v=3, L=2)	0.33 (1.20)	0.37 (1.49)	0.29 (1.64)	0.34 (1.64)	0.42 (2.29)	0.52 (2.89)	0.47 (3.02)	0.48 (3.02)
MACE-H (G, v=3, L=5)	0.32 (1.25)	0.37 (1.40)	0.28 (1.69)	0.33 (1.69)	0.38 (2.37)	0.43 (2.41)	0.46 (3.38)	0.43 (3.38)
MACE-H (G, v=1, L=1)	0.35 (1.39)	0.39 (1.57)	0.29 (1.54)	0.35 (1.59)	0.40 (2.07)	0.46 (2.33)	0.46 (4.04)	0.45 (4.04)

Table S8: The MAE (in meV) on Au data using DeepH-E3, MACE-H with/without shift-and-scale operation. The values in the parentheses show the maximum error of the Hamiltonian matrix elements.

	DeepH-E3	MACE-H	MACE-H (Shift & Scale)
Au	0.41 (0.80)	0.32 (0.65)	0.27 (0.62)

Table S9: MAE (in meV) on bilayer graphene without SOC and Bismuthene with SOC using different settings of radial basis and many body expansion and its relation to the locality. Values in parentheses show the maximum error of the Hamiltonian matrix elements. The B and G in parentheses stand for Bessel and Gaussian radial basis set, respectively.

model	graphene (noSOC)		Bismuthene (SOC)	
	shifted	twisted	shifted	twisted
DeepH-E3	0.40 (0.73)	0.28 (1.04)	0.26 (1.55)	0.40 (3.94)
MACE-H (B, v=3, L=5)	0.29 (0.52)	0.24 (0.78)	0.25 (1.4)	0.52 (6.29)
MACE-H (G, v=3, L=5)	0.31 (0.51)	0.24 (0.80)	0.23 (1.27)	0.45 (4.79)
MACE-H (G, v=1, L=1)	0.40 (0.75)	0.24 (0.59)	0.24 (1.50)	0.37 (3.39)

Table S10: Summary of employed datasets.

Dataset	system dimension	DFT package	number of layers	SOC	data size (train/val/test)	system size (atoms)
graphene	2D	OpenMx	monolayer	noSOC	450 (0.6/0.2/0.2)	72
MoS_2	2D	OpenMx	monolayer	noSOC	500 (0.6/0.2/0.2)	75
graphene	2D	OpenMx	bilayer	noSOC	300 (0.6/0.2/0.2)	64
bismuthene	2D	OpenMx	bilayer	SOC	576 (0.4/0.2/0.2)	36
Bi_2Se_3	2D	OpenMx	bilayer	SOC	576 (0.4/0.2/0.2)	90
Bi_2Te_3	2D	OpenMx	bilayer	noSOC	256 (0.8/0.15/0.05)	90
Bi_2Te_3	2D	OpenMx	bilayer	SOC	256 (0.8/0.15/0.05)	90
Au	Bulk	FHI-aims	/	noSOC	200 (0.76/0.19/0.05)	64

Table S11: Radial cutoffs of numeric atom-centered orbitals (NAOs) in the employed datasets. The values can be used to infer the sparsity of the Hamiltonian and, therefore, the resulting connectivity graph.

	C	Mo	S	Bi	Se	Te	Au
Radius	6.0 a_0	7.0 a_0	7.0 a_0	8.0 a_0	7.0 a_0	7.0 a_0	5.0 Å
